# Reliability, validity, and effectiveness of center of pressure parameters in assessing stabilometric platform in subjects with incomplete spinal cord injury: a serial cross-sectional study

**DOI:** 10.1186/1743-0003-11-86

**Published:** 2014-05-13

**Authors:** Federica Tamburella, Giorgio Scivoletto, Marco Iosa, Marco Molinari

**Affiliations:** 1 Clinical and Movement Analysis Research Laboratory- CaRMA Lab, Spinal Cord Unit, IRCCS Santa Lucia Foundation, Rome, Italy; 2 Clinical Laboratory of Experimental Neurorehabilitation, IRCCS Santa Lucia Foundation, Rome, Italy

**Keywords:** Spinal cord injury, Stabilometry, Center of pressure, Balance, Reliability, Validity

## Abstract

**Background:**

Spinal cord injury (SCI) can damage long tracts, affecting postural stability. Impairments in balance have recently been proposed to be highly predictive of functional recovery in patients with SCI and thus merit evaluation. In addition to common observational clinical scales, more objective evaluation methods of balance can be implemented by analyzing center of pressure (COP) parameters using stabilometric platforms (SPs). COP analysis has been used in various pathologies, but the COP parameters with regard to measurement vary, depending on the features of the target population, and have only been assessed in healthy subjects. Specifically, concerning subjects with SCI, few studies have reported COP parameters, and none has addressed the reliability, validity, or responsiveness of this measure. The objective of this serial cross-sectional study was to analyze the reliability, validity, and responsiveness of COP parameters under various conditions in incomplete SCI subjects to assess balance.

**Methods:**

Twenty-three patients with incomplete SCI were examined 111 times for 1 year. Each session comprised administration of the Berg Balance scale, Tinetti scale, and WISCI scale and evaluation of stabilometric platform use. Stabilometry was performed under various sensory conditions (OF: open feet; CF: closed feet; OE: open eyes; CE: closed eyes), wherein several COP parameters were analyzed (L: COP path length; V: mean COP velocity, V_AP:_ anteroposterior COP velocity; V_LL_: laterolateral COP velocity, A: COP ellipse area, SA1: x-axis of COP ellipse area; SA2: y-axis of COP ellipse area). The reliability, validity, and responsiveness of COP parameters that were associated with visual/support area conditions were analyzed.

**Results:**

Of the COP parameters, V and arithmetically related measures had the highest reliability, validity, and effectiveness scores. Of all test conditions, OE-OF was the most valid, whereas CE-OF was the most responsive.

**Conclusion:**

The assessment of balance in SCI subjects can be reliable, valid, and effective in acquiring V data, based on OF-OE and OF-CE conditions and heel distance values.

## Background

Balance is usually defined as preservation of the vertical projection of the body’s center of mass (COM) onto the support area that is formed by the feet [1]. Human balance is typically modeled as an inverted pendulum, in which the body is controlled as a single rigid segment that supports a single mass point—the COM—which rotates around the ankle joint [2]. The inverted pendulum is regulated through the development of ground-reaction forces, the vector sum of which is applied to a point that is defined as the vertical projection of the COM onto the ground [3]: the center of pressure (COP).

The body’s equilibrium is maintained by the central nervous system, which fixes the COM around a specific point—a goal that is under constant challenge by continuous perturbations to the COM by factors, such as breathing, heart rate, and muscle activity [4]. To maintain postural stability, several afferent inputs, such as visual, vestibular, and somatosensory, are integrated and converted into efferent motor outputs, which in turn are transmitted down to the spinal cord along various motor tracts [5]. Postural sway, such as spontaneous shifts in the COP during quiet standing, represents the integrated output of complex interactions between systems [6]. Damage to any of these systems can result in postural instability, affecting static and dynamic balance—ie, stance and gait [7]. Of the postural control systems, the spinal centers have a significant function, explaining the clinical relevance of postural control deficits in spinal cord injury (SCI) [8,9].

Despite the availability of many technical instruments to assess balance, the most common clinical tools remain observational scales, such as the Tinetti [10] and Berg balance scales [11]. Nevertheless, these scales are hampered by a lack of sensitivity and objectivity and are limited by floor-ceiling effects [11,12].

To overcome these drawbacks, stabilometric platforms (SPs), consisting of a rigid plate that is supported by force transducers, and COP analyses have been introduced in clinical settings [6]. Many studies have reported the use of various SPs to evaluate balance deficits in healthy subjects [13] and in several pathologies, including orthopedic diseases [14], neuropathic lesions [15], essential tremor [16], Parkinson disease [15], multiple sclerosis [17], muscular dystrophy [18], cerebral palsy [19], cerebellar ataxia [20], and stroke [21]. Two recent studies assessed balance in SCI, examining recoveries after visual biofeedback rehabilitation by COP analysis [8,9]. Impaired balance is a significant limitation to overground ambulation in patients with SCI [11], and impairments in balance are predictive of gait recovery [22], thus meriting evaluation [9].

Despite the growing interest in balance, the standardization of COP parameters with regard to measurements and the related quality domains [23,24] (ie, reliability, validity, and responsiveness) [25] is poor [26] and is absent from the SCI population. COP measurements have been examined in healthy elderly individuals [6,27] and in patients with Parkinson [28] and orthopedic diseases [29]. Data from healthy subjects can inflate the reliability estimates, because measurements can be made in them more easily than in patients [6].

Measurement errors, and hence the reliability of a measure, are not fixed but depend on the study population [30] and can vary between test conditions [6]. Thus, measurement properties must be specified for a study population and test conditions.

No study has examined the properties of COP parameters by SP in subjects with SCI. Our serial cross-sectional study aimed to determine the reliability, validity, and responsiveness of COP parameters under various test conditions and define the protocol parameters that are suitable for specifically assessing balance in subjects with incomplete motor SCI.

## Methods

### Population

This serial cross-sectional study included 23 subjects with incomplete motor SCI. The inclusion criteria comprised traumatic and nontraumatic etiology, subacute and chronic AIS D SCI, and the ability to maintain a standing position unsupported for at least 52 s. The exclusion criteria were the presence of cognitive impairments and any orthopedic or neurological pathology that could influence the assessment of balance. Neurological status was scored per American Spinal Injury Association (ASIA) standards, including the Impairment Scale (AIS) [31]. Patients’ demographics, lesion levels, and etiologies are reported in Table 1.

**Table 1:** Patients’ clinical and epidemiological features

	**Age**	**Gender**	**Weight**	**Height**	**Aethiology**	**Lesion level**	**Time since lesion (months)**	**OF-OE**	**OF-CE**	**CF-OE**	**CF-CE**
PT1*	19	M	62	173	T	T7	6	Y	Y	N	N
PT2*	34	F	68	175	NT (Inflammatory)	C5	24	Y	Y	Y	Y
PT3*	66	M	74	167	NT (Degenerative)	T11	15	Y	Y	Y	Y
**PT4**	37	M	64	171	T	C6	13	Y	Y	Y	Y
PT5*	52	M	68	169	NT (Vascular)	T12	10	Y	Y	Y	Y
PT6*	33	F	55	167	T	T11	8	Y	Y	N	N
PT7*	34	F	60	176	NT (Vascular)	T8	6	Y	Y	Y	Y
PT8*	54	F	70	168	NT (Degenerative)	L5	32	Y	Y	Y	N
PT9*	35	F	66	172	NT (Degenerative)	L4	8	Y	N	Y	N
**PT10**	41	M	88	177	T	L3	5	Y	Y	N	N
PT11*	64	M	78	160	NT (Inflammatory)	T5	13	Y	Y	N	N
**PT12**	84	M	53	165	NT (Inflammatory)	L1	8	Y	Y	N	N
**PT13**	52	M	80	173	NT (Degenerative)	C7	8	Y	Y	Y	Y
PT14*	30	M	65	173	T	L3	9	Y	Y	Y	Y
**PT15**	40	M	73	178	T	L3	6	Y	Y	Y	Y
PT16*	29	M	65	181	T	T10	8	Y	N	N	N
PT17*	61	F	80	159	NT (Inflammatory)	T7	14	Y	N	N	N
PT18*	33	F	85	182	T	C6	8	Y	Y	Y	Y
PT19*	59	F	60	158	NT (Degenerative)	C5	72	Y	Y	Y	Y
PT20*	69	M	75	165	T	C5	13	Y	Y	Y	Y
PT21*	44	F	57	178	NT (Degenerative)	D1	75	Y	Y	Y	Y
PT22*	51	M	74	173	NT	C7	9	Y	Y	Y	Y
**PT23**	60	M	65	170	NT (Degenerative)	C7	8	Y	Y	Y	Y
***Medium (s.d.)***	*48,27 (15,94)*	*14 M - 9 F*	*69,22 (9,37)*	*163,45 (32,65)*	*60.9% NT 39.1% T*		*16.43 (19,03)*				

T: traumatic lesion; NT: nontraumatic lesion; Lesion level: C: cervical; T: thoracic; L: lumbar. Sensory test conditions: OF: open feet; CF: closed feet; OE: open eyes; CE: closed eyes; Y: assessment performed; N: assessment not performed.*Patients with SCI who underwent at least 2 consecutive balance assessments, both clinical and instrumental.

Enrolled patients were assessed repeatedly. Specifically, 6 patients were evaluated once, and the remaining 17 patients were assessed 2 to 12 times for 1 year, with 2 weeks between sessions. Overall, 111 evaluation sessions were analyzed, all of which administered clinical and instrument-based assessments of balance. The local ethics committee approved this study (Prot. CE/AG.4-PROG.231-65), and all patients gave informed consent for participation.

### Clinical assessment of balance

For each evaluation, the Berg Balance scale (BBS) and Tinetti scale (TS) were used to assess balance clinically, and the Walking Index for Spinal Cord Injury (WISCI) [32] was used to determine the functional level of ambulation. The BBS is a 14-item task-oriented test that was validated recently in SCI patients [11] and can be considered a reflection of functional activity. Total scores range from 0 to 56, with higher scores indicating greater balance and functional independence. The TS includes subscores for equilibrium (TS_E_) and locomotion (TS_L_) [10]. Fourteen items on this clinical test measure balance characteristics (scored out of 24), and 10 items examine gait features (scored out of 16), for a total score of 40, with higher scores indicating greater balance. The WISCI has been validated specifically with regard to gait in subjects with SCI [33]. Total scores range from 0 to 20, with higher scores reflecting greater independent locomotion [32].

### Instrument-based stabilometric assessment of balance

Stabilometric parameters were analyzed using a 320-cm by 75-cm (length x width) static force platform (Platform BPM 120, Physical Support Italia, Italy). The signals were amplified and acquired using dedicated software (Physical gait Software Vv. 2.66, Physical Support Italia, Italy). In assessing static stability, patients stood barefoot in a natural and relaxed position with their arms by their sides and with both heels lined up [6], under 2 sensory conditions: eyes open and facing a target 1.5 m away (OE) and eyes closed (CE).

The feet were placed with the forefoot open 30 degrees and the heels in 2 positions: together (FT) or apart at a comfortable distance (FA). For the FA condition, heel distance (HD) was measured manually by the operator and fixed for the FA-OE and FA-CE conditions and during the recordings. For each evaluation, 4 conditions (FT-OE, FT-CE, FA-OE, and FA-CE) were tested. Under each condition, measures were recorded 3 times, per Ruhe [6]. We selected 51.2 s as the testing time, per the platform manufacturer and other studies [9,34,35]. A slight pause was permitted between recordings to allow the arms to rest on bars, during which the patients were asked to maintain their foot position on the platform. During the data collection, subjects were asked to “stand as still as possible” while looking straight ahead, per Zok et al. [36].

We considered the following quantitative COP parameters:

– Length indicators: path length (L, mm), mean velocity (V, L divided by the trial duration), anteroposterior (V_AP_) and laterolateral (V_LL_) velocities (mm/s), and mean position of COP along the planar laterolateral (X) and anteroposterior (Y) coordinates on the platform (mm).

– Surface indicators: area of the ellipse encompassing 90% of COP samples (A, cm^2^) and length of its semiaxes (SA_1_, SA_2_, cm).

### Data analysis

#### Demographic features

The influence of demographic features on COP parameters was analyzed using the Spearman correlation coefficient (ρ), applied to data from the first session of each subject.

#### Measurement of COP parameters between test conditions

The *reliability* domain contains various measures for continuous data [23]: test-retest and intrarater reliability, measurement error, and minimal detectable change.

$$\%\mathrm{MD}C_{95}=\left(\mathrm{MD}C_{95}*100\right)/\mathrm{baseline}\;\mathrm{assessment}\;\mathrm{value}.$$

1. *Test-retest reliability,* or *repeatability,* reflects the reliability between tests by the same operator in the same session. Test-retest reliability was assessed using the coefficient of variation (CV). CV is a measure of data dispersion and was the standard deviation that was computed for the values in the 3 recordings, expressed as a percentage of the mean value. Because the ideal mean for planar coordinates is 0, CV was not computed for the Y or X COP.

2. *Intrarater reliability* determines the reliability across the time of evaluations by the same operator. For continuous data, intraclass correlation (ICC) is the preferred method [23], because it also takes systematic errors between repeated measurements into account. Of the various methods of calculating ICC, consistent with the Shrout and Fleiss reliability coefficients guidelines [37], we adopted the ICC(3,1) form. We analyzed the ICC, with a confidence interval (CI) of 95%, for patients who underwent at least 2 consecutive (within 15 days) assessments (17 subjects).

3. *Measurement error* indicates the absolute error in measurement and was calculated as the standard error of measurement (SEM) [23]. SEM represents the standard deviation of repeated measures of the same subject (ie, within-subject variability) by the same operator (ie, within-rater variability) and is expressed in units of the measurement tool—in this case, SEM = SD * √ (1-ICC).

4. *Minimal detectable change* (MDC_95_) addresses the common problem of deciding whether results are significant or due to errors in measurement. MDC_95_ is defined as the minimal amount of change that is not due to the variation in measurement [38]. Calculated as SEM *1.65 * √2, MDC_95_ determines the magnitude of change that exceeds the threshold of measurement error at a 95% confidence level. A 95% confidence interval, as with SEM, increases the precision of score estimates [38]. Further, the percentage of MDC_95_ that indicates the percentage of the minimal amount by which the results change versus baseline—not due to variations in measurement—is calculated per the following formula:

Reliability assessments were also performed for the BBS, TIN, and WISCI using ICC, SEM, and MDC_95_.

The *validity* domain refers to the degree to which an instrument measures the construct that it purports to measure [23] and is evaluated based on criterion and construct validity.

1. C*riterion validity* indicates the degree to which the scores of a measurement instrument are an adequate reflection of a standard. The preferred method for estimating criterion validity is correlation coefficient, which should preferably exceed 0.70 [23]. For patients with SCI, the only validated tool for assessing balance is the BBS [11], rendering it the standard tool for determining criterion validity by Spearman correlation coefficient (ρ).

2. C*onstruct validity* estimates the consistency of measurement instrument scores under the assumption that the instrument measures the construct validity [23], which is calculated as *convergent validity*. Construct validity refers to the degree to which COP parameters correlate with the related scales (BBS and TS_E_)—not with other scales (WISCI and TS_L_) (ie, *appropriateness*). Convergent validity was analyzed using correlation coefficients (R for Pearson coefficient and ρ for Spearman coefficient).

The *responsiveness* domain reflects the sensitivity to changes and is frequently measured by effect size (ES) [39]. ES is based on the data distribution and is the mean difference between values in the first and second assessments, divided by the standard deviation of the baseline values (ie, the values in the first assessment). ES was calculated for patients who participated in at least 2 sessions (17 patients) with regard to clinical and instrumental data.

#### Effects of sensory conditions on assessment

The optimal sensory conditions for balance assessment were analyzed by analysis of variance (ANOVA), with vision and support base as the main factors. Further, Pearson correlation between HD and clinical scales was analyzed to determine the influences of HD on balance in SCI subjects.

Statistical analyses were performed using SPSS for Windows (version 9.0, Chicago, IL). Data were considered significant at p < 0.05. Correlation analyses were performed per Munro’s classification [30]: 0.00–0.25: little, if any correlation; 0.26–0.49: low correlation; 0.50–0.69: moderate correlation; 0.70–0.89: high correlation; and 0.90–1.00: very high correlation.

## Results

Not all subjects were tested under each sensory condition; subjects with more severe damage were unable to perform the most challenging tasks; CF or CE conditions. Overall, 111 OF-OE, 96 OF-CE, 83 CF-OE, and 73 CF-CE evaluations were performed.

By Spearman correlation analysis, no significant correlations between COP parameter and demographic features (age, height, weight, gender) were observed for any sensory condition (OE, CE, OF, CF) (p > 0.05).

### Measurement properties of COP parameters between test conditions

With regard to reliability*, test-retest reliability* was evaluated by CV for each COP parameter in 3 trials under each condition. The CV of all parameters was minimally affected by sensory condition, indicating their lack of effect on the reliability COP parameters. The most repeatable parameters—those with the lowest CV values—were L, V, and V_LL_. The least repeatable parameter was A, with a mean CV of approximately 50% over all conditions (Table 2).

**Table 2:** Test-retest reliability of COP parameters by coefficient of variation

**Parameter**	**Coefficient of Variation [%]**				
	**OF-OE**	**OF-CE**	**CF-OE**	**CF-CE**	**Mean (SD)**
**A**	46.4	46.7	47.2	42.4	*45.7 (2.2)*
**L**	13.4	13.6	13.8	12.9	***13.4 (0.3)***
**SA1**	27.7	24.9	22.6	26.0	*25.3 (2.1)*
**SA2**	27.5	26.3	27.8	25.1	*26.7 (1.2)*
**V**	13.3	13.6	13.8	12.9	***13.4 (0.4)***
**V**_**LL**_	13.2	13.3	12.7	14.2	***13.4 (0.6)***
**V**_**AP**_	16.2	15.6	18.0	13.3	*15.8 (1.9)*
**Mean (SD)**	*22.5 (12.3)*	*22.0 (12.2)*	*22.3 (12.3)*	*21.0 (11.1)*	

The assessment conditions and mean coefficient of variation (CV) for each COP parameter between the 3 trials are reported. The mean values between conditions for each COP parameter (last column) or COP parameters for each condition (last row) are in italic characters. Bold faced numbers in the last column identify the lowest CV values among COP parameters.

The *intrarater reliability* was assessed by ICC for the clinical scales and COP parameters. Of the clinical scales, the BBS had the highest ICC value, whereas the TIN had the lowest. For COP parameters, averaged between sensory conditions, L, V, and V_LL_ had the highest ICC values. In contrast, with regard to sensory conditions, averaged between COP parameters, the OF-OE and OF-CE conditions had the highest ICC values, whereas the CF-CE condition had the lowest ICC (Table 3). The ICC value of the BBS was the highest of all clinical scales and COP parameters.

**Table 3:** **Intrarater reliability of COP parameters by ICC, SEM, MDC**_**95**_**, and%MDC**

**Scale**	**ICC**	**SEM**	**MDC**_**95**_	**%MDC**																
**BBS**	0,97***	2,07	5,74	17,2																
**TIN**	0,22	3,54	9,81	58,5																
**TIN**_**E**_	0,87***	0,86	2,37	26,3																
**TIN**_**L**_	0,78***	1,07	2,97	38,3																
**WISCI**	0,95**	0,73	0,02	13,0																
**COP**	**OF-OE**				**OF-CE**				**CF-OE**				**CF-CE**				***Mean (sd)***			
**Parameter**	**ICC**	**SEM**	**MDC**_**95**_	**%MDC**	**ICC**	**SEM**	**MDC**_**95**_	**%MDC**	**ICC**	**SEM**	**MDC**_**95**_	**%MDC**	**ICC**	**SEM**	**MDC**_**95**_	**%MDC**	**ICC**	**SEM**	**MDC**_**95**_	**%MDC**
**A**	0,64**	1,67	4,63	141,9	0,94***	2,33	6,46	74,6	0,92***	0,89	2,47	69,0	0,65*	5,62	15,58	*116,9*	*0,79 (0,71)*	*2,63*	*7,29*	*100,6*
**L**	0,89***	37,23	103,2	46,5	0,92***	67, 27	186,46	49,6	0,92***	21,75	60,29	27,2	0,74**	105,53	292,5	*55,5*	*0,87 (0,08)*	*57,95*	*160,62*	***44,8***
**SA1**	0,85***	0,34	0,94	64,7	0,81***	0,49	1,34	72,1	0,88***	0,22	0,62	43,3	0,79*	0,69	1,93	*61,0*	*0,83 (0,04)*	*0,44*	*1,21*	*60.2*
**SA2**	0,71***	0,44	1,21	71,5	0,89***	0,54	1,5	58,0	0,76**	0,39	1,08	58,9	0,38	6,17	17,09	*796,0*	*0,68 (0,22)*	*1,88*	*5,22*	*246,1*
**Y**	**0,7**	2,48	6,88	255,5	0,34	5,47	15,17	466,4	0,37	3,36	9,32	413,3	0,54*	3,9	10,09	*189,0*	*0,49 (0,16)*	*3,8*	*10,55*	*331,0*
**X**	0,54*	2,87	7,96	1297,2	0,34	2,35	6,52	1552,0	0,01	2,68	7,42	769,5	0,28	3,55	9,83	*18189,0*	*0,29 (0,22)*	*2,86*	*7,93*	*5451,9*
**V**	0,89***	0,72	2,01	46,3	0,92***	1,31	3,64	49,9	0,92***	0,43	1,18	27,4	0,74*	2,06	5,7	*55,4*	***0,87 (1,13)***	*1,13*	*3,13*	***44,8***
**V**_**LL**_	0,89***	0,48	1,32	43,9	0,9***	0,73	2,03	47,9	0,9***	0,3	0,82	28,9	0,83***	1,14	3,15	***49,2***	***0,88 (0,03)***	*0,66*	*1,83*	***42,5***
**V**_**AP**_	0,82***	0,59	1,64	65,0	0,89***	1,22	3,39	66, 7	0,82***	0,44	1,21	**45,1**	0,65*	1,65	4,57	*68,4*	*0,79 (0,1)*	*0,97*	*2,70*	*61,3*
*Mean*	*0,77*	*5,20*	*14,42*		*0,77*	*9,08*	*25,17*		*0,72*	*3,38*	*9,38*		*0,62*	*14,48*	*40,13*					
*(SD)*	*0,13*	*12,05*	*33,39*		*0,13*	*21,88*	*60,64*		*0,32*	*6,98*	*19.35*		*0,19*	*34,20*	*94,80*					

Significant ICC data are in bold (p < 0.05:*, p < 0.005:**, p < 0.001:***). The mean ICC, SEM, MDC95, and %MDC values between conditions for each COP parameter (last column) or COP parameters for each condition (last row) are in italic characters. The highest mean ICC and %MDC values are in bold faced characters. For abbreviations of COP parameters and sensory conditions, see list of abbreviations.

Due to differences between unit measures, percentage change in MDC_95_ between sessions was used instead of MDC_95_ for the statistical analysis. Of all clinical scales, the BBS and WISCI had the lowest percentage change due to measurement error, and L, V, and V_LL_ had the lowest percentage change due to measurement error of all COP parameters (Table 3).

*Validity* of the COP parameters was evaluated by correlation analysis with clinical scales, as reported in Table 4. Of the COP parameters, L, V, V_LL,_ and V_AP_ correlated significantly and systematically with the BBS, TS, and TS_E_. L, V, and V_LL_ also correlated with the TSL but only under the OF-OE/CE conditions, with low R values. Notably, the highest correlations between COP parameters and clinical scales were observed with each parameter in the OF-OE condition. X and Y data did not correlate with the clinical scales (Table 4).

**Table 4:** Validity of COP parameters

**Coefficient of correlation**		**Clinical scales**					
		**BBS**	**TS**	**TS-E**	**TS-L**	**WISCI**	**mean(|R|)**
**A**	**OF-OE**	- 0.65***	−0.49***	−0.57***	−0.23*	−0.09	0.41
	**OF-CE**	−0.48***	−0.27**	−0.42***	0.07	−0.14	0.28
	**CF-OE**	−0.55***	−0.44***	−0.52***	−0.25*	−0.08	0.37
	**CF-CE**	−0.31***	−0.17	−0.26***	−0.02	−0.16	0.18
**L**	**OF-OE**	−0.73***	−0.58***	−0.64***	−0.23*	−0.21*	0.48
	**OF-CE**	−0.54***	−0.34***	−0.43***	0.06	−0.21*	0.32
	**CF-OE**	−0.61***	−0.54***	−0.54***	−0.23*	−0.16	0.42
	**CF-CE**	−0.39***	−0.24***	−0.32***	0.01	−0.39***	0.27
**SA1**	**OF-OE**	−0.68***	−0.53***	−0.61***	−0.30**	−0.11	0.45
	**OF-CE**	−0.52***	−0.34***	−0.47***	0.02	−0.19	0.31
	**CF-OE**	−0.46***	−0.40***	−0.43***	−0.18	−0.09	0.31
	**CF-CE**	−0.26*	−0.20	−0.23*	−0.10	−0.27*	0.21
**SA2**	**OF-OE**	−0.66***	−0.48***	−0.57***	−0.21*	−0.09	0.40
	**OF-CE**	−0.50***	−0.30**	−0.42***	0.02	−0.06	0.26
	**CF-OE**	−0.58***	−0.44***	−0.53***	−0.23*	−0.05	0.37
	**CF-CE**	−0.11	0.00	−0.11	0.07	−0.06	0.07
**Y**	**OF-OE**	−0.02	−0.08	−0.08	−0.05	0.22*	0.09
	**OF-CE**	−0.13	−0.13	−0.14	−0.13	0.02	0.11
	**CF-OE**	0.02	−0.12	−0.08	−0.18	0.29*	0.14
	**CF-CE**	−0.09	−0.03	−0.04	−0.05	−0.31*	0.10
**X**	**OF-OE**	0.00	0.10	0.04	0.01	−0.13	0.06
	**OF-CE**	−0.01	0.04	0.01	0.17	−0.11	0.07
	**CF-OE**	−0.13	0.00	−0.10	−0.05	−0.18	0.09
	**CF-CE**	−0.17	−0.15	−0.12	−0.01	−0.14	0.12
**V**	**OF-OE**	−0.73***	−0.58***	−0.64***	−0.23*	−0.22*	0.48
	**OF-CE**	−0.52***	−0.30**	−0.41***	0.10	−0.24*	0.31
	**CF-OE**	−0.61***	−0.54***	−0.54***	−0.23*	−0.16	0.42
	**CF-CE**	−0.39**	−0.24*	−0.32**	0.01	−0.39*	0.27
**V**_**LL**_	**OF-OE**	−0.71***	−0.60***	−0.64***	−0.30**	−0.22*	0.50
	**OF-CE**	−0.54***	−0.34***	−0.44***	0.04	−0.27*	0.32
	**CF-OE**	−0.52***	−0.49***	−0.47***	−0.26*	−0.18	0.38
	**CF-CE**	−0.35**	−0.24*	−0.29*	−0.07	−0.39***	0.27
**V**_**AP**_	**OF-OE**	−0.74***	−0.56***	−0.64***	−0.18	−0.20*	0.47
	**OF-CE**	−0.53***	−0.28*	−0.40***	0.16	−0.19	0.31
	**CF-OE**	−0.69***	−0.56***	−0.59***	−0.20	−0.13	0.44
	**CF-CE**	−0.41***	−0.24*	−0.32**	0.07	−0.35**	0.28

Statistically significant values are in bold (p < 0.05:*, p < 0.005:**, p < 0.001:***). The mean values of absolute values of these coefficients between conditions are reported in last column.

Our evaluation of *criterion validity* by correlation of COP parameters with the BBS determined L, V, V_LL,_ and V_AP_ in the OF-OE condition to be the only parameters with R values over the validity criterion of 0.70. *Convergent validity* was assessed by comparing COP parameters with balance- and nonbalance-related clinical scales. The correlation coefficients of the COP parameters were higher for balance scale scores (BBS, TS, TS_E_) versus locomotion scale scores (TS_L_, WISCI).

The evaluation of *responsiveness,* as measured by ES, is shown in Table 5. The BBS was the most sensitive clinical scale, with an ES of 0.78. All COP parameters were more sensitive than the clinical scales, with nearly all ES values above 1. Averaging between sensory conditions, L, V, V_LL_, and V_AP_ had the highest ES values. For the sensory conditions, averaged between COP parameters, the OF-OE and CF-CE conditions had the highest ES values (Table 5).

**Table 5:** Responsiveness of COP parameter assessment

	**Effect size (ES)**				
**Scale**					
**BBS**	**0.78**				
**TIN**	0.18				
**TIN**_**E**_	0.37				
**TIN**_**L**_	0.17				
**WISCI**	O.07				
**Parameter**	**OF-OE**	**OF-CE**	**CF-OE**	**CF-CE**	**Mean (SD)**
**A**	2.10	0.81	1.80	1.69	*1.60 (0.55)*
**L**	1.87	2.41	1.39	2.96	***2.16 (0.68)***
**SA1**	1.28	1.73	0.58	2.93	*1.63 (0.98)*
**SA2**	2.72	0.91	1.25	1.05	*1.48 (0.84)*
**Y**	1.36	2.29	1.53	1.43	*1.65 (0.43)*
**X**	1.52	1.08	1.11	0.49	*1.05 (0.51)*
**V**	1.87	2.41	1.39	2.96	***2.16 (0.68)***
**V**_**LL**_	2.79	3.33	1.13	1.90	***2.29 (0,97)***
**V**_**AP**_	2.60	1.39	2.56	2.88	***2.36 (0.66)***
**Mean (SD)**	*2.01 (0.58)*	*1.82 (0.85)*	*1.42 (0.54)*	*2.03 (0.98)*	

Effect size (ES) between 2 sessions for 17 SCI patients. The mean values between conditions for each COP parameter (last column) or between COP parameters for each condition (last row) are in italic characters. The highest ES values among the clinical scales and COP parameters are in bold.

### Effects of assessments of sensory conditions

**Figure 1:**
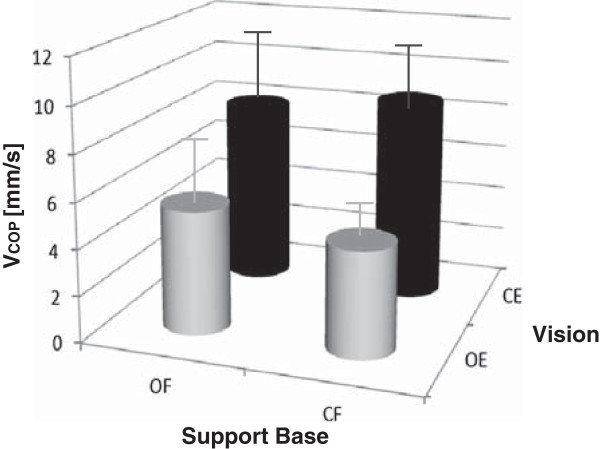
**Effects of conditions on stabilometry.** Histograms of mean COP velocity (V ± standard deviation) versus assessment conditions, depending on support base [open feet (OF) versus closed feet (CF)] and vision [open eyes (OE) versus closed eyes (CE)].

Considering the data above, the effects of sensory conditions were examined, focusing on V—the most sensitive, reliable, and valid COP parameter (Figure 1). Overall, foot position had little effect on V, whereas vision affected V significantly. By ANOVA of V values, with vision and support base as the main effects, only vision had a significant effect [F(1.346) = 76.10; vision: p < 0.001, support base: p = 0.535, interaction not significant: p = 0.445.] The COP data were lower for the OE versus CE condition, indicating better balance with vision (Figure 1). The lack of a support base effect suggests that the foot conditions have little influence. These data are consistent with the lack of an effect of foot position on COP parameters or scale score correlation data (Table 4).

**Figure 2:**
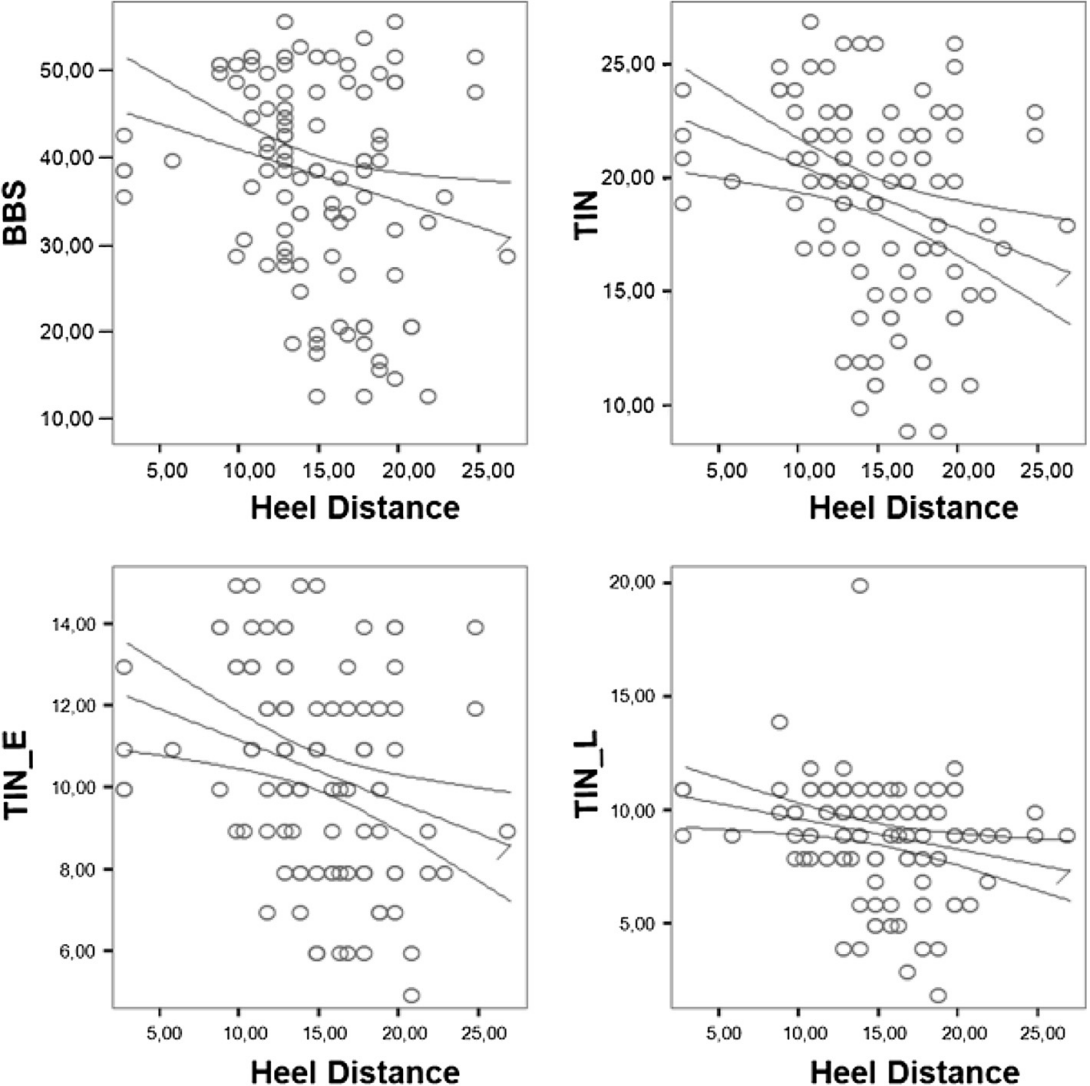
**Correlation analysis between heel distance and clinical scales.** Correlation between HD and BBS, TIN, TIN_E_, and TIN_LOC_.

In contrast, in the OF condition, HD was self-selected, and patients used disparate HD conditions. By Pearson correlation analysis between HD and clinical scales, the BBS (ρ = −0.227, p = 0.019), TIN_E_ (ρ = −0.275, p = 0.003), and TIN (ρ = −0.289, p = 0.03) were significant, but TIN_L_ was not (x = −0.112, p = 0.24) (Figure 2).

The importance of the HD in assessing balance with regard to balance recovery was analyzed longitudinally for 17 patients who underwent rehabilitation by considering the HD during the evaluations over time. The mean R was high (R = −0.37, p < 0.001), indicating a progressive reduction in HD of 3 cm for 150 days in patients who were assessed repeatedly.

## Discussion

Reliability, validity, and responsiveness are the key components of determining the suitability of a measurement; these parameters can vary by characteristic of the target population [30]. Our data allow us to define the COP parameters and assessment conditions that are suitable in evaluating postural balance in subjects with incomplete SCI.

**Demographic features** have been claimed to affect the reliability of COP measurements [6]. Although few studies have addressed the effect of gender, all of them agree in disputing sex-related influences on balance, consistent with our findings [40,41]. Height and weight affect COP parameters [42,43]. In our study, we adopted the approach of Salavati et al. [29] in mitigating the effects of height and weight by averaging COP measurements between 3 consecutive trials. Consequently, no correlations between height or weight data and COP parameters were observed.

The influence of age is debated [40,44]. In our cohort, there were no significant correlations between COP parameter and age, supporting the hypothesis that age does not affect SP evaluations in the SCI population. Nevertheless, our study did not specifically aim to determine the effects of aging, and no specific measures were taken to correctly evaluate confounding factors.

### Measurement properties of COP parameters between test conditions

*Reliability* can be measured in several ways, of which ICC is the most common in SP studies [6]. Independent of method (CV or ICC) and sensory condition (OF-OE, OF-CE, CF-OE, CF-CE), L, V, and V_LL_ were the most reliable parameters. L is directly (ie, arithmetically) related to V and recording time, and L and V provide the same information, if the recording time has been standardized [13]. Thus, all V and L measures should be considered related, because they are based on the same raw values. The reliability of V in SCI is consistent with findings on V in examining balance in young healthy [6] and old healthy [27] subjects and in patients with orthopedic diseases [29]. Current studies agree that V is the most reliable parameter in assessing balance.

When examining a patient population and possible treatment effects, the significance of a detected change—ie, whether a change is reliable or due to variations in measurement—must be determined [6]. This step is commonly addressed in clinical studies by SEM and MDC_95_[27,29], the latter of which is most frequently used in SCI [45-47]. Notably, of the COP parameters, V and the related COP parameters L and V_LL_ had the lowest MDC_95_ scores.

As discussed and consistent with Scoltes et al. [23], in addition to reliability, the quality of the instruments’ measurements must be established by considering validity and responsiveness—2 parameters that are seldom reported in SP studies. *Validity* is established by reference to the gold standard measurement in this field and by taking into account that the instrument measures the desired construct. BBS is the only balance scale that has been validated specifically for the SCI population [11]. Thus, we analyzed *criterion validity* by correlation analysis between BBS and COP parameters.

When dealing with validity measures, correlation coefficients that exceed 0.70 are considered significant [23]; in our data, V, L, V_LL_, and V_AP_ were the only measures that had correlation coefficients above 0.70. High V validity was also evidenced by the results on convergent validity. Our comparison of correlation coefficients between V and the related COP data and clinical scale—with the same (BBS and TS_E_) or different (TS_L_ and WISCI) constructs—demonstrated high correlation values for BBS and TS_E_ but not for TS_L_ or WISCI. Overall, these findings implicate V as a valid parameter in assessing balance in SCI subjects. Such evaluation methods are unavailable for healthy subjects and other clinically relevant populations. Thus, the validity of V in populations other than SCI patients must be determined.

*Responsiveness* is the ability of an instrument to detect changes in the construct that is measured over time [48]. Whereas validity refers to a single score, responsiveness reflects the validity of a score that has changed [48]. There is an ongoing debate about the ideal method for evaluating responsiveness [49,50]. It has been suggested that all responsiveness measures are measures of longitudinal validity or treatment effects and that, specifically for responsiveness, assessing longitudinal validity should be the preferred method [49,50]. However, no longitudinal validity assessment tool is available for SP. Based on the limitations that were discussed recently by Mokkink et al. [49], we used the most common method, ES, in evaluating responsiveness in SCI subjects. Consistent with previous domains, higher responsiveness was recorded for V and the related L, V_LL_, and V_AP_ measures.

It could be argued that a high intersession ICC score is inconsistent with high intersession responsiveness, unless all subjects undergo similar changes between sessions, as was the case in our cohort—all patients had well-stabilized clinical profiles. Thus, very few changes were expected and recorded in the 2-week intersession period.

### Effects of sensory condition assessments

In a recent review, Ruhe et al. reported the absence of standardized methods for COP measurements and implicated trial duration, repetition, and visual and foot conditions as critical factors for obtaining reliable COP datasets [6]. Attempts to provide recommendations on the length and number of trials for assessing balance correctly have failed to reach a consensus.

*Trial duration* varies between studies. The recommended trial duration is 90 to 120 s to effect acceptable reliability with correlation coefficients > 0.75 for most parameters [6]. Nevertheless, early studies reported that a 10–60-sec duration was suitable for obtaining reliable COP data [51,52]. In our study, we did not examine the influence of differences in time on the reliability of COP parameters. The recording time was set to 51.2 per the platform handbook. Although it was short compared with recent recommendations [6], this duration yielded high correlation coefficients (>0.70) for most parameters.

With regard to *trial repetitions*, there is a tendency to increase trial number to generate more reliable data. Although this pattern might be reasonable when examining young healthy subjects, it becomes impractical when recruiting disabled persons in a clinical setting. Thus, we did not determine trial repetition effects and set a low number of repetitions to permit averaging (3, per Ruhe) [6].

*Vision effects* on the reliability of COP measures have been evaluated in several studies on population-related effects [6]. Recent studies have reported a trend toward higher reliability estimates under the eyes-closed conditions, prompting the recommendation to keep the eyes closed as the best practice. Our study subjects were tested with the eyes open (OE) and closed (CE), although not all subjects were able to perform under the CE condition, indicating the CE condition to be more challenging and discriminating. The significant effect of vision on V values also highlighted its significance.

There were no differences between the 2 visual conditions with regard to the reliability of V. Conversely, vision affected the validity and responsiveness of V indexes. The highest validity was obtained under the OE condition, and the best responsiveness was seen under the CE conditions. The discrepancy between the effects of vision on the validity and responsiveness of V and the lack of an effect of vision on the reliability of V is notable and contrasts the findings of Ruhe [6], suggesting that the eyes-closed condition should be applied, at least in healthy subjects. Validity and responsiveness refer to 2 different domains—ie, validity evaluates the construct that it purports to measure, whereas responsiveness reflects the sensitivity to patient changes. Thus, V can be evaluated with and without vision, at least in SCI subjects.

*Foot position* affects passive stability, decreasing the request of active neural control [42,53], but no consensus exists on the more reliable foot position [6]. Despite this lack of normative data, the best practice guidelines suggest standardization [6]. We tested subjects under 2 feet conditions: CF and OF. In the OF condition, subjects were asked to stand comfortably with their heels apart, and HD was recorded. This setting allowed us to test the closed versus open conditions and determine the effects of HD on COP reliability.

In general, a narrow stance is at least as reliable as a comfortable stance [6], but our findings indicate that foot position (CF or OF) does not affect the reliability of COP parameters. Conversely, the OF condition allowed us to record HD, for which the balance scales had high correlation values. The significance of changes in HD over time suggests that HD can be used to evaluate the effects of recovery and treatment on balance. Overall, OF in the comfortable position with HD recorded appears to be the ideal test condition for SCI subjects, consistent with the recommendations of Chiari [42] and Yoon [40] for healthy subjects.

Based on the limitations above, taking into account the V data, OF-OE is the most valid condition and OF-CE is the most responsive condition, suggesting that both should be implemented in testing SCI subjects.

**Figure 3:**
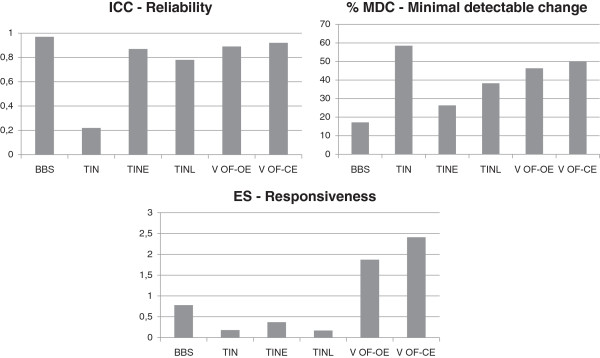
**Comparison of ICC,%MDC, and ES results between balance scales and V data for OF-OE and OF-CE conditions.**

The comparison of reliability and responsiveness between the V value of COPs in the OF-OE and OF-CE conditions and the balance scales merits further examination. To facilitate the comparison between the V and balance scale results, the ICC, %MDC, and ES data are presented in Figure 3. Because BBS is the reference standard, the V results only approximate the BBS ICC data. Yet, greater changes in V versus BBS in patient balance are required to obtain improvements that are not due to instrument error.

The most notable comparison concerns responsiveness. V ES, particularly in the OF-CE condition, was superior to all clinical scales. The difference between ES values was 0.78 for BBS and 2.41 for V in the OF-CE condition. Thus, the proposed SP protocol significantly increases the ability to detect changes in the balance of SCI subjects compared with BBS.

Our study did not specifically evaluate the reliability, validity, and effectiveness of SP parameters for evaluating recovery after SCI. This aspect must be addressed in a devoted study, with repeated measures conducted during rehabilitation and balance recovery.

Limitations: Instructions to the patients, time of testing, and trial repetitions were present and were not tested experimentally. The reliability, validity, and effectiveness of COP parameters in assessing stabilometric platform were not been tested in healthy control subjects’ group. The psychometric properties of COP measurements in healthy individuals have been examined in several studies [6], particularly in the elderly [27,44]. Nevertheless, the assessment of detailed data on healthy controls should be included in a devoted study.

## Conclusion

To obtain a reliable and valid instrument for assessing balance by SP in SCI patients, COP V data must be acquired, based on OF-OE and OF-CE conditions, reporting heel distance values for OF conditions.

## Abbreviations

SCI: Spinal cord injury; COP: Center of pressure; COM: Center of mass; SP: Stabilometric platform; ASIA: American spinal injury association; AIS: ASIA impairment scale; BBS: Berg balance scale; TS: Tinetti scale; TSE: Tinetti equilibrium; TSL: Tinetti scale locomotion; WISCI: Walking index for spinal cord injury; OF: Open feet; CF: Closed feet; OE: Open eyes; CE: Closed eyes; HD: Heel distance; L: COP path length; V: Mean COP velocity; VAP: Anteroposterior COP’s velocity; VLL: Laterolateral COP velocity; A: COP ellipse area; SA1: X-axis of COP ellipse area; SA2: Y-axis of COP ellipse area; CV: Coefficient of variation; ICC: Intraclass correlation coefficient; CI: Confidence interval; SEM: Standard error of measurement; MDC95: Minimal detectable change; ES: Effect size.

## Competing interests

Authors declare that they have no competing interests.

## Authors’ contributions

F.T.: conceived of the study, participated in its design, acquired and collected data, performed interpretation of data and statistical analysis, and wrote the manuscript. G.S.: conceived of the study, participated in its design, and helped draft the manuscript. M.I: participated in the design of the study and helped in the statistical analysis. M.M.: participated in the design of the study, wrote the manuscript, and gave the final approval. All authors have read and approved the final manuscript.

## References

[B1] Kingma I Toussaint HM Commissaris DA Savelsbergh GJ Adaptation of center of mass control under microgravity in a whole-body lifting task Exp Brain Res 1999 125 35 42 10.1007/s002210050655 10100974

[B2] Winter DA Patla AE Prince F Ishac M Gielo-Perczak K Stiffness control of balance in quiet standing J Neurophysiol 1998 80 1211 1221 9744933 10.1152/jn.1998.80.3.1211

[B3] Winter DA Prince F Frank JS Powell C Zabjek KF Unified theory regarding A/P and M/L balance in quiet stance J Neurophysiol 1996 75 2334 2343 8793746 10.1152/jn.1996.75.6.2334

[B4] Carpenter MG Murnaghan CD Inglis JT Shifting the balance: evidence of an exploratory role for postural sway Neuroscience 2010 171 196 204 10.1016/j.neuroscience.2010.08.030 20800663

[B5] Liechti M Muller R Lam T Curt A Vestibulospinal responses in motor incomplete spinal cord injury Clin Neurophysiol 2008 119 2804 2812 10.1016/j.clinph.2008.05.033 18842452

[B6] Ruhe A Fejer R Walker B The test-retest reliability of centre of pressure measures in bipedal static task conditions–a systematic review of the literature Gait Posture 2010 32 436 445 10.1016/j.gaitpost.2010.09.012 20947353

[B7] Pokorna K Use of stabilometric platform and visual feedback in rehabilitation of patients after the brain injury Prague Med Rep 2006 107 433 442 17402556

[B8] Sayenko DG Alekhina MI Masani K Vette AH Obata H Popovic MR Nakazawa K Positive effect of balance training with visual feedback on standing balance abilities in people with incomplete spinal cord injury Spinal Cord 2010 48 12 886 893 doi: 10.1038/sc.2010.41. Epub 2010 Apr 20 10.1038/sc.2010.41 20404833

[B9] Tamburella F Scivoletto G Molinari M Balance training improves static stability and gait in chronic incomplete spinal cord injury subjects: a pilot study Eur J Phys Rehabil Med 2013 49 353 364 23486301

[B10] Tinetti ME Williams TF Mayewski R Fall risk index for elderly patients based on number of chronic disabilities Am J Med 1986 80 429 434 10.1016/0002-9343(86)90717-5 3953620

[B11] Lemay JF Nadeau S Standing balance assessment in ASIA D paraplegic and tetraplegic participants: concurrent validity of the Berg Balance Scale Spinal Cord 2010 48 245 250 10.1038/sc.2009.119 19773797

[B12] Rosenfeld J Jackson CE BKatirji HJKDCP Quantitativeassessment and outcomemeasures in neuromuscular disease Neuromuscular Disorders in Clinical Practice 2002 1 309 343

[B13] Raymakers JA Samson MM Verhaar HJ The assessment of body sway and the choice of the stability parameter(s) Gait Posture 2005 21 48 58 10.1016/j.gaitpost.2003.11.006 15536033

[B14] Lopez-Rodriguez S de-Las-Penas Fernandez C Alburquerque-Sendin F Rodriguez-Blanco C Palomeque-del-Cerro L Immediate effects of manipulation of the talocrural joint on stabilometry and baropodometry in patients with ankle sprain J Manipulative Physiol Ther 2007 30 186 192 10.1016/j.jmpt.2007.01.011 17416272

[B15] Schieppati M Tacchini E Nardone A Tarantola J Corna S Subjective perception of body sway J Neurol Neurosurg Psychiatry 1999 66 313 322 10.1136/jnnp.66.3.313 10084529 PMC1736274

[B16] Bove M Marinelli L Avanzino L Marchese R Abbruzzese G Posturographic analysis of balance control in patients with essential tremor Mov Disord 2006 21 192 198 10.1002/mds.20696 16161140

[B17] Karst GM Venema DM Roehrs TG Tyler AE Center of pressure measures during standing tasks in minimally impaired persons with multiple sclerosis J Neurol Phys Ther 2005 29 170 180 10.1097/01.NPT.0000282314.40230.40 16388684

[B18] Aprile I Padua L Iosa M Gilardi A Bordieri C Frusciante R Iannaccone E Erra C Padua L Balance and walking in facioscapulohumeral muscular dystrophy: multiperspective assessment Eur J Phys Rehabil Med 2012 48 393 402 22713540

[B19] Donker SF Ledebt A Roerdink M Savelsbergh GJ Beek PJ Children with cerebral palsy exhibit greater and more regular postural sway than typically developing children Exp Brain Res 2008 184 363 370 10.1007/s00221-007-1105-y 17909773 PMC2137946

[B20] Morton SM Bastian AJ Relative contributions of balance and voluntary leg-coordination deficits to cerebellar gait ataxia J Neurophysiol 2003 89 1844 1856 12612041 10.1152/jn.00787.2002

[B21] Nardone A Godi M Grasso M Guglielmetti S Schieppati M Stabilometry is a predictor of gait performance in chronic hemiparetic stroke patients Gait Posture 2009 30 5 10 10.1016/j.gaitpost.2009.02.006 19318253

[B22] Scivoletto G Romanelli A Mariotti A Marinucci D Tamburella F Mammone A Cosentino E Sterzi S Molinari M Clinical factors that affect walking level and performance in chronic spinal cord lesion patients Spine 2008 33 259 264 10.1097/BRS.0b013e3181626ab0 18303457

[B23] Scholtes VA Terwee CB Poolman RW What makes a measurement instrument valid and reliable? Injury 2011 42 236 240 10.1016/j.injury.2010.11.042 21145544

[B24] Diehr P Chen L Patrick D Feng Z Yasui Y Reliability, effect size, and responsiveness of health status measures in the design of randomized and cluster-randomized trials Contemp Clin Trials 2005 26 45 58 10.1016/j.cct.2004.11.014 15837452

[B25] Karanicolas PJ Bhandari M Kreder H Moroni A Richardson M Walter SD Norman GR Guyatt GH Evaluating agreement: conducting a reliability study J Bone Joint Surg Am 2009 91 Suppl 3 99 106 19411507 10.2106/JBJS.H.01624

[B26] Terwee CB Bot SD de Boer MR van der Windt DA Knol DL Dekker J Bouter LM De Vet HC Quality criteria were proposed for measurement properties of health status questionnaires J Clin Epidemiol 2007 60 34 42 10.1016/j.jclinepi.2006.03.012 17161752

[B27] Moghadam M Ashayeri H Salavati M Sarafzadeh J Taghipoor KD Saeedi A Salehi R Reliability of center of pressure measures of postural stability in healthy older adults: effects of postural task difficulty and cognitive load Gait Posture 2011 33 651 655 10.1016/j.gaitpost.2011.02.016 21458272

[B28] Rocchi L Chiari L Cappello A Horak FB Identification of distinct characteristics of postural sway in Parkinson's disease: a feature selection procedure based on principal component analysis Neurosci Lett 2006 394 140 145 10.1016/j.neulet.2005.10.020 16269212

[B29] Salavati M Hadian MR Mazaheri M Negahban H Ebrahimi I Talebian S Sanjari MA Sohani SM Parniapour M Test-retest reliability [corrected] of center of pressure measures of postural stability during quiet standing in a group with musculoskeletal disorders consisting of low back pain, anterior cruciate ligament injury and functional ankle instability Gait Posture 2009 29 460 464 10.1016/j.gaitpost.2008.11.016 19167891

[B30] Domholdt E Rehabilitation Research: Principles and Applications 2005 3 Philadelphia: Elsevier Saunders

[B31] American Spinal Injury Association International Standard for Neurological Classification of Spinal Cord Injury (rev) 2000 Chicago: American Spinal Injury Association 1 23

[B32] Ditunno PL Dittuno JF Jr Walking index for spinal cord injury (WISCI II): scale revision Spinal Cord 2001 39 654 656 10.1038/sj.sc.3101223 11781863

[B33] Ditunno JF Jr Barbeau H Dobkin BH Elashoff R Harkema S Marino RJ Hauck WW Apple D Basso DM Behrman A Deforge D Fugate L Saulino M Scott M Chung J Validity of the walking scale for spinal cord injury and other domains of function in a multicenter clinical trial Neurorehabil Neural Repair 2007 21 539 550 10.1177/1545968307301880 17507642 PMC4080923

[B34] Isableu B Fourre B Vuillerme N Giraudet G Amorim MA Differential integration of visual and kinaesthetic signals to upright stance Exp Brain Res 2011 212 33 46 10.1007/s00221-011-2693-0 21533556

[B35] Giovanni A Akl L Ouaknine M Postural dynamics and vocal effort: preliminary experimental analysis Folia Phoniatr Logop 2008 60 80 85 10.1159/000114649 18235195

[B36] Zok M Mazza C Cappozzo A Should the instructions issued to the subject in traditional static posturography be standardised? Med Eng Phys 2008 30 913 916 10.1016/j.medengphy.2007.12.002 18243033

[B37] Shrout PE Fleiss JL Intraclass correlations: uses in assessing rater reliability Psychol Bull 1979 86 420 428 18839484 10.1037//0033-2909.86.2.420

[B38] Steffen T Seney M Test-retest reliability and minimal detectable change on balance and ambulation tests, the 36-item short-form health survey, and the unified Parkinson disease rating scale in people with parkinsonism Phys Ther 2008 88 733 746 10.2522/ptj.20070214 18356292

[B39] Portney LG Watkins MP Portney LG, Watkins MP Responsiveness to change. Foundations of clinical research: applications to practice Prentice Hall Health 2000 2 New Jersey, USA 103 105

[B40] Yoon JJ Yoon TS Shin BM Na EH Factors affecting test results and standardized method in quiet standing balance evaluation Ann Rehabil Med 2012 36 112 118 10.5535/arm.2012.36.1.112 22506243 PMC3309333

[B41] Hageman PA Leibowitz JM Blanke D Age and gender effects on postural control measures Arch Phys Med Rehabil 1995 76 961 965 10.1016/S0003-9993(95)80075-1 7487439

[B42] Chiari L Rocchi L Cappello A Stabilometric parameters are affected by anthropometry and foot placement Clin Biomech (Bristol, Avon ) 2002 17 666 677 10.1016/S0268-0033(02)00107-9 12446163

[B43] Hue O Simoneau M Marcotte J Berrigan F Dore J Marceau P Tremblay P Teasdale N Body weight is a strong predictor of postural stability Gait Posture 2007 26 32 38 10.1016/j.gaitpost.2006.07.005 16931018

[B44] Demura S Kitabayashi T Noda M Aoki H Age-stage differences in body sway during a static upright posture based on sway factors and relative accumulation of power frequency Percept Mot Skills 2008 107 89 98 18986036 10.2466/pms.107.1.89-98

[B45] Scivoletto G Tamburella F Laurenza L Molinari M The spinal cord independence measure: how much change is clinically significant for spinal cord injury subjects Disabil Rehabil 2013 35 21 1808 1813 doi: 10.3109/09638288.2012.756942. Epub 2013 Jan 24 10.3109/09638288.2012.756942 23343359

[B46] Scivoletto G Tamburella F Laurenza L Molinari M Distribution-based estimates of clinically significant changes in the international standards for neurological classification of spinal cord injury motor and sensory scores Eur J Phys Rehabil Med 2013 49 373 384 23486305

[B47] Burns AS Delparte JJ Patrick M Marino RJ Ditunno JF The reproducibility and convergent validity of the Walking Index for Spinal Cord Injury (WISCI) in chronic spinal cord injury Neurorehabil Neural Repair 2011 25 149 157 10.1177/1545968310376756 21239706

[B48] Mokkink LB Terwee CB Patrick DL Alonso J Stratford PW Knol DL Bouter LM De Vet AC The COSMIN study reached international consensus on taxonomy, terminology, and definitions of measurement properties for health-related patient-reported outcomes J Clin Epidemiol 2010 63 737 745 10.1016/j.jclinepi.2010.02.006 20494804

[B49] van der Heijden GJ Leffers P Bouter LM Shoulder disability questionnaire design and responsiveness of a functional status measure J Clin Epidemiol 2000 53 29 38 10.1016/S0895-4356(99)00078-5 10693901

[B50] Wilson IB Cleary PD Linking clinical variables with health-related quality of life. A conceptual model of patient outcomes JAMA 1995 273 59 65 10.1001/jama.1995.03520250075037 7996652

[B51] Schmid M Conforto S Camomilla V Cappozzo A D'Alessio T The sensitivity of posturographic parameters to acquisition settings Med Eng Phys 2002 24 623 631 10.1016/S1350-4533(02)00046-2 12376049

[B52] Le CK Riach C Postural stability measures: what to measure and for how long Clin Biomech (Bristol, Avon) 1996 11 176 178 10.1016/0268-0033(95)00027-5 11415618

[B53] Henry SM Fung J Horak FB Effect of stance width on multidirectional postural responses J Neurophysiol 2001 85 559 570 11160493 10.1152/jn.2001.85.2.559

